# G4 Structures in Control of Replication and Transcription of rRNA Genes

**DOI:** 10.3389/fpls.2020.593692

**Published:** 2020-10-08

**Authors:** Kateřina Havlová, Jiří Fajkus

**Affiliations:** ^1^ Laboratory of Functional Genomics and Proteomics, National Centre for Biomolecular Research, Faculty of Science, Masaryk University, Brno, Czechia; ^2^ Chromatin Molecular Complexes, Mendel Centre for Plant Genomics and Proteomics, Central European Institute of Technology, Masaryk University, Brno, Czechia; ^3^ Department of Cell Biology and Radiobiology, Institute of Biophysics of the Czech Academy of Sciences, Brno, Czechia

**Keywords:** rDNA stability, transcription, replication, quadruplex DNA, G4, ribosomal RNA genes

## Abstract

Genes encoding 45S ribosomal RNA (rDNA) are known for their abundance within eukaryotic genomes and for their unstable copy numbers in response to changes in various genetic and epigenetic factors. Commonly, we understand as epigenetic factors (affecting gene expression without a change in DNA sequence), namely DNA methylation, histone posttranslational modifications, histone variants, RNA interference, nucleosome remodeling and assembly, and chromosome position effect. All these were actually shown to affect activity and stability of rDNA. Here, we focus on another phenomenon – the potential of DNA containing shortly spaced oligo-guanine tracts to form quadruplex structures (G4). Interestingly, sites with a high propensity to form G4 were described in yeast, animal, and plant rDNAs, in addition to G4 at telomeres, some gene promoters, and transposons, suggesting the evolutionary ancient origin of G4 as a regulatory module. Here, we present examples of rDNA promoter regions with extremely high potential to form G4 in two model plants, *Arabidopsis thaliana* and *Physcomitrella patens*. The high G4 potential is balanced by the activity of G4-resolving enzymes. The ability of rDNA to undergo these “structural gymnastics” thus represents another layer of the rich repertoire of epigenetic regulations, which is pronounced in rDNA due to its highly repetitive character.

## Introduction

Among many potential reasons to become interested in genes encoding ribosomal RNA (rRNA) is the possibility to study the wide range of regulatory mechanisms used to control their expression and genomic stability. When starting from the genomic level, genes for 45S rRNA (rDNA) usually form the most abundant gene family in most eukaryotes (e.g., 150 copies per haploid genome in *Saccharomyces cerevisiae*, Kobayashi et al., 1998; 300 in human, Schmickel, 1973; Agrawal and Ganley, 2018; and 600 in *Arabidopsis thaliana*, Pruitt and Meyerowitz, 1986; Copenhaver et al., 1995) with a considerable individual variability in a copy number. Variability can also be seen in the lengths and nucleotide sequences of intergenic spacers separating individual transcription units of 18S-5.8S-25S transcribed by RNA Polymerase I, while the nucleotide sequences of genes coding for 18S, 5.8S and 25S rRNAs are highly conserved (reviewed in Dvorackova et al., 2015). rDNAs form one or more tandemly arranged gene clusters (nucleolus organizing regions, NORs) per haploid genome whose sizes are maintained within a standard range as a result of dynamic balance between the loss and recovery of individual rDNA repeats. rDNA copies are most notably lost by intra-chromatid recombination between distant rDNA copies, leading to excision of the intervening copies in the form of an extrachromosomal rDNA circle. These events can be counteracted by various recombination events, e.g., an unequal sister chromatid recombination or an unequal sister chromatid exchange, which are induced in a response to DNA double strand breaks generated due to arrested replication forks (see Nelson et al., 2019, for recent review).

In some organisms, e.g., *S. cerevisiae* (Bayev et al., 1980) or the moss *Physcomitrella patens* (Goffova et al., 2019), rDNA units also comprise 5S rRNA genes inserted in the intergenic spacers between individual 18S-5.8S-25S transcription units. 5S rRNA is not present in the primary RNA Pol I transcript but is transcribed by RNA Pol III. Besides RNA Pol I and – in some cases – RNA Pol III promoters, intergenic spacers also show the presence of additional promoters (spacer promoters), which may promote transcription by RNA Pol I or II, giving rise to non-coding (nc)RNAs affecting rRNA expression (Doelling et al., 1993; Mayer et al., 2006; Cesarini et al., 2010; Earley et al., 2010; Agrawal and Ganley, 2018).

Indeed, rDNA clusters represent a miniature system of their own where concurrent functions of different kinds of promoters and polymerases can be observed, replication origins are present (and obviously closely spaced), replication and transcription polymerases can meet and occasionally collide, and DNA repair mechanisms must eventually solve problems arising from all this apparent turmoil.

On the other hand, this mini-world has also developed numerous tools of precise regulation which began to be understood in molecular details recently. These include a phenomenon termed nucleolar dominance (see, e.g., Preuss and Pikaard, 2007; Chandrasekhara et al., 2016; Mohannath et al., 2016).

Further, the importance of an appropriate higher order chromatin arrangement for rDNA stability was highlighted in recent studies (Pontvianne et al., 2013, 2016), as well as was the role of histone chaperones in the assembly of the very basic units of chromatin – the nucleosomes (Mozgova et al., 2010; Muchova et al., 2015; Pavlistova et al., 2016). Further, the role of DNA methylation and histone acetylation in the control of rDNA activity has been elucidated (Probst et al., 2004; Grummt, 2007; Mcstay and Grummt, 2008; Pontvianne et al., 2010; Schmitz et al., 2010), as well as the enigmatic importance of keeping a considerable fraction of rDNA units inactive (Kobayashi, 2011).

In addition to all the interesting knowledge accumulated on rDNA/rRNA topics in the last decades, a specific feature of rDNA has been observed – its propensity to form tetraplex (quadruplex) structures (G4), which are based on guanine tetrads. This feature seems to be conserved throughout eukaryotes (Hanakahi et al., 1999; Hershman et al., 2008; Capra et al., 2010; Goffova et al., 2019; Matyasek et al., 2019; Mestre-Fos et al., 2019a) and is thought to contribute significantly to the inherently low stability of rDNA as an obstacle to advancing replication forks. Stalled and collapsed replication forks then induce repair events which may result in rDNA loss or expansion (see above). Effects of the high propensity to form G4 structures become more evident when functions of intrinsic factors (e.g., specific helicases) which are able to dissolve G4 structures are disrupted or compromised, resulting in a hyper-recombinogenic character of rDNA and its instability.

Here, we exemplify the role of G4 structures in rDNA of two model plants, *P. patens* and *A. thaliana*.

## Destabilization of RDNA Due to Dysfunction of G4-Resolving Helicases and Colocalization of G4 Sites With Gene and Spacer Promoters in *Arabidopsis Thaliana* RDNA

In *Arabidopsis thaliana*, it was found recently that RecQ-mediated genome instability protein 2 (RMI2) and Regulator of telomere elongation helicase 1 (RTEL1) contribute to the stability of the 45S rDNA copy number (Rohrig et al., 2016). RMI2 in *Arabidopsis*, as well as in yeasts and humans, acts for a proper dissolution of recombination intermediates, thereby suppressing a hyper-recombinogenic phenotype (Wu and Hickson, 2003). Also, RTEL1 (initially described in *Caenorhabditis elegans*) functions as a Fe-S cluster helicase suppressing inappropriate recombination events by promoting disassembly of D-loop recombination. Furthermore, RTEL1 can dissolve quadruplex (G4) DNA structures that otherwise block the extension of telomeres by telomerase (Vannier et al., 2012), and in humans, its dysfunction causes Hoyeraal-Hreidarsson syndrome, a severe form of dyskeratosis congenita, which is characterized by short telomeres and genome instability (Le Guen et al., 2013; Vannier et al., 2013, 2014; Faure et al., 2014). RTEL1 also promotes genome-wide replication through its interaction with PCNA, increasing replication fork stability, extension rates, and origin usage (Vannier et al., 2013).

Both AtRMI2 and AtRTEL1 participate in the maintenance of rDNA stability in parallel pathways. In *atrmi2* plants, 45S rDNA decreased to 80%, in *atrtel1* plants to 40%, and in double *atrmi2 atrtel1* mutants to ca. 30% of their standard copy number (Rohrig et al., 2016). A similar contribution to rDNA stability was also observed in another Fe-S cluster helicase – FANCJ homolog in *Arabidopsis* – AtFANCJB (Dorn et al., 2019).

**Figure 1:**
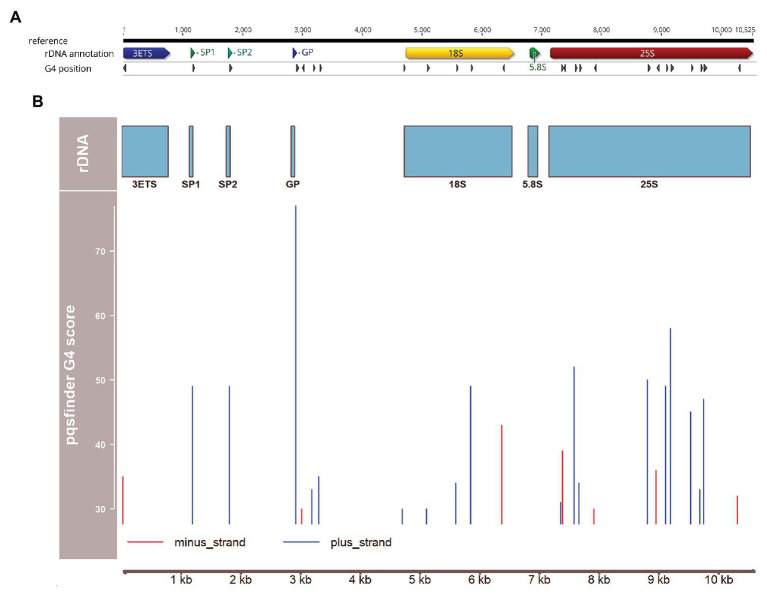
Distribution of potential G4-forming sequences over the 45S ribosomal DNA (rDNA) unit of *Arabidopsis thaliana*. **(A)** Map of the rDNA unit using the data from Chandrasekhara et al. (2016), and the Geneious software platform (Biomatters, Auckland, New Zealand). Positions of 3' external transcribed Spacer (3ETS), spacer promoter 1 and 2 (SP1, SP2, respectively), gene promoter (GP) and 18S, 5.8S, and 25S rRNA genes are indicated. **(B)** Positions and scores of G4 structures predicted using pqsfinder (Hon et al., 2017) and plotted with the Bioconductor package Gviz (Hahne and Ivanek, 2016).

These results are consistent with the fact that *A. thaliana* rDNA repeat units show the presence of a cluster of sites with a strong potential to form a G4 structure (Figure 1). The highest score obtained using the pqsfinder tool (Hon et al., 2017; Labudova et al., 2020) coincides with the gene promoter (GP) site, reaching a value (77) higher than the scores of the best characterized G4-forming DNAs, plant or human telomeric repeats (60 and 64, respectively; Goffova et al., 2019). Presumably, formation of G4 in the plus-strand at the promoter sites may strongly inhibit 45S rDNA transcription and slow down its replication. Two other G4 sites were detected at spacer promoters, SP1 and SP2. Interestingly, the number of spacer promoters (and, consequently, a number of G4s) varies among rDNA units in *A. thaliana* (Havlova et al., 2016), which may represent a novel layer in regulation of transcription and replication of individual rDNA units. Yet, additional G4 sites were found inside the coding regions for 18S rRNA and 25S rRNA (Figure 1). These results are thus consistent with the view that G4 sites play important roles not only in rDNA replication and genome stability (supported by the abovementioned observations on *A. thaliana* helicase mutants) but also in control of rDNA transcription.

## In Addition to the Features Observed in *Arabidopsis Thaliana*, a Cluster of G4 Sites Separates 5s and 18s RRNA Genes Transcribed With pol III and pol I, Respectively, in *Physcomitrella Patens*

The situation in *P. patens* rDNA is complicated by the linked arrangement between 18S-5.8S-25S units and 5S rRNA genes. This arrangement has been demonstrated recently (Goffova et al., 2019) and is congruent with its earlier description in a liverwort, *Marchantia polymorpha*, and a moss *Funaria hygrometrica* (Sone et al., 1999), as well as with a later systematic study in land plants (Wicke et al., 2011). *P. patens RTEL1* mutants (*pprtel1*), similar to *atrtel1* mutants, also show a marked decrease of 18S rDNA copies (representing 45S rDNA), but, in addition, a comparable decrease of 5S rDNA is observed (Goffova et al., 2019). Interestingly, while reduced relative transcript levels of 18S rRNA roughly correspond to the decrease in their genomic copies in *pprtel1* plants, reduction in 5S rRNA transcripts is more pronounced, without any obvious relation to 5S rDNA copy number. This indicates a relatively independent regulation of 5S and 45S rDNA transcription.

**Figure 2:**
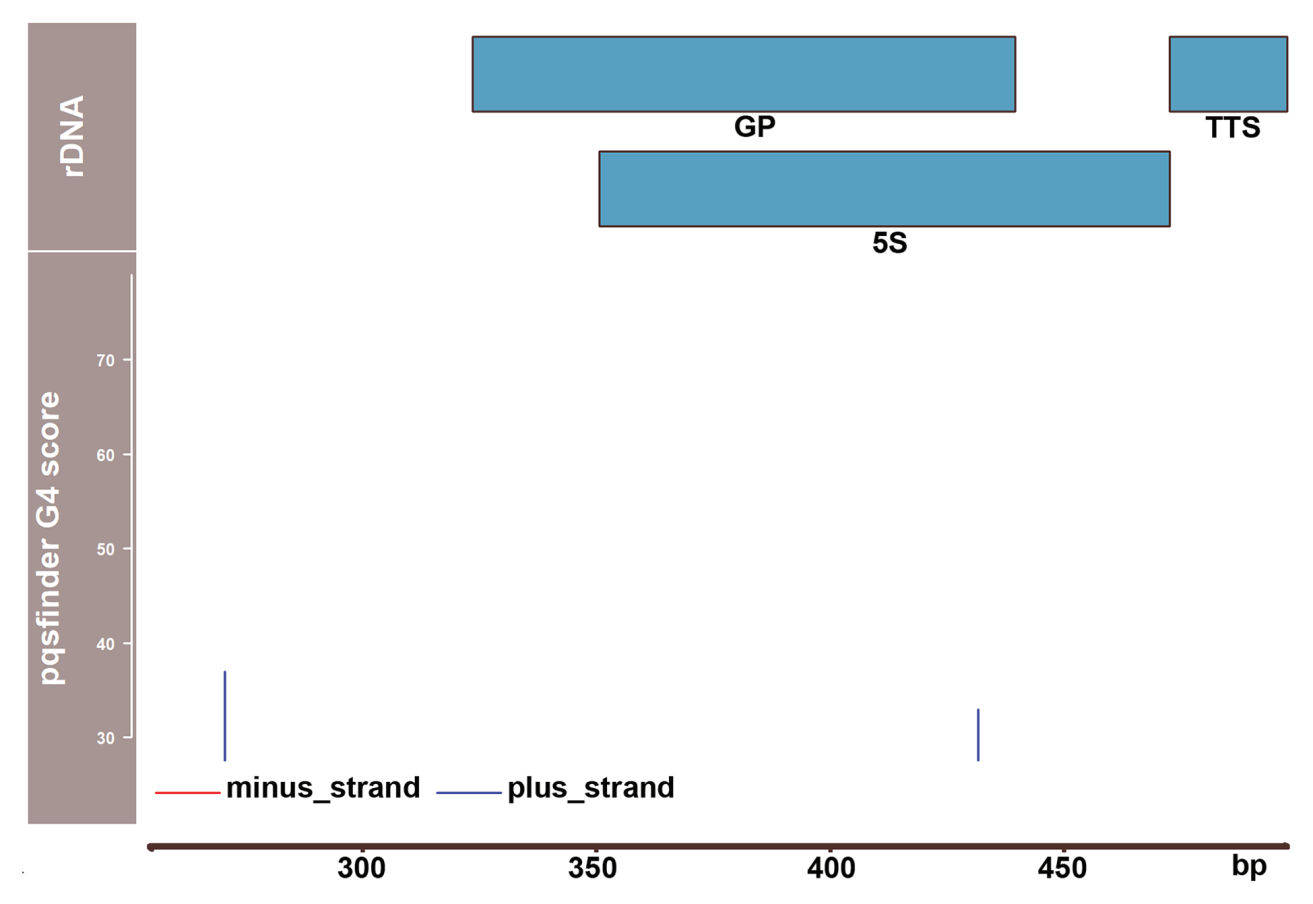
Distribution of potential G4-forming sequences over the 5S rDNA unit of *A. thaliana*. 5S rDNA sequence data (Campell et al., 1992) were used to analyze pqsfinder scores. Two putative G4 sites starting in positions 257 and 414 were identified with pqsfinder showing the scores of 37 and 33, respectively. The GP including both external and internal elements, 5S rRNA gene (5S) and transcription termination site (TTS) are depicted using the data from Cloix et al. (2003).

In a search of a mechanistic explanation of our results, we found a noticeable clustering of putative G4 sites in the spacer region between 5S and 18S rRNA genes (Goffova et al., 2019). Prediction of G4 propensity revealed a particularly strong site in the plus-strand (thus with a presumable inhibitory role in transcription) ca. 500 bp upstream of the 18S rRNA gene where the pqsfinder score reached a value of 132, which is twice higher than that of telomere DNA. These results were confirmed by another prediction tool, G4Hunter (Bedrat et al., 2016). An independent indication of the high G4 potential of this region was supported by our observation that PCR amplification was problematic across the linker between the 5S and 18S rRNA genes, requiring addition of dimethyl sulfoxide (DMSO) to the reaction mixture. Furthermore, our repeat clustering analysis indicated a high potential of this region to form non-canonical structures by a dramatically (two orders of magnitude) lower number of NGS reads when compared with the neighboring regions (Goffova et al., 2019). Thus, it is conceivable that in addition to the G4 roles suggested in *A. thaliana* rDNA based on experiments and predictions, yet another putative function is provided by G4 sites in *P. patens* – a protection against collision or interference between advancing RNA Polymerases I and III. This hypothesis is supported by the absence of any sites of a comparable G4 potential in *A. thaliana* 5S rDNA unit, which is located separately from the 45S rDNA locus (Figure 2).

## Conclusion

G4 formation and resolution can be regarded as a dynamic switch whose identity is defined genetically – through its primary DNA sequence – but its “ON” and “OFF” states are controlled by the local availability of G4-targeting proteins or other ligands that affect the G4 stability positively or negatively. As this switch acts in control of transcription and replication without a change in the primary DNA sequence, we suggest that the formation of G4 structures (and possibly also the other relevant non-canonical DNA secondary structures) be included among epigenetic mechanisms.

In rDNA, epigenetic effects of G4 formation can be expected preferentially at active copies (where a lesser nucleosome density or even nucleosome removal can be expected around transcription start sites – thereby facilitating formation of G4) or during replication when DNA strands are temporarily separated and noncanonical intrastrand structures can be formed. In addition to G4s formed by rDNA as discussed above, recent results suggest possible roles of G4s formed by rRNAs. Interestingly, these potential G4s are located on surfaces of both subunits of the human ribosome (Mestre-Fos et al., 2019b). When assuming that rRNA is the most abundant fraction of cellular RNA, then these G4-rRNAs clearly dominate the total population of RNA quadruplexes, thus indicating another perspective topic of future studies.

## Data Availability Statement

All datasets presented in this study are included in the article.

## Author Contributions

KH performed annotation of rDNA region and prediction of G4 sites. JF wrote the manuscript. Both authors have read, edited, and approved the final version of the manuscript.

### Conflict of Interest

The authors declare that the research was conducted in the absence of any commercial or financial relationships that could be construed as a potential conflict of interest.
